# Custom Design of Packaging through Advanced Technologies: A Case Study Applied to Apples

**DOI:** 10.3390/ma12030467

**Published:** 2019-02-03

**Authors:** Lucía Rodríguez-Parada, Pedro F. Mayuet, Antonio J. Gámez

**Affiliations:** Department of Mechanical Engineering and Industrial Design, University of Cadiz, 11519 Cadiz, Spain; pedro.mayuet@uca.es (P.F.M.); antoniojuan.gamez@uca.es (A.J.G.)

**Keywords:** food packaging, customization, product design, personalized design, reverse engineering, computer aid design (CAD), fused deposition modelling (FDM)

## Abstract

In the context of food packaging design, customization enhances the value of a product by meeting consumer needs. Personalization is also linked to adaptation, so the properties of the packaging can be improved from several points of view: functional, aesthetic, economic and ecological. Currently, functional and formal properties of packaging are not investigated in depth. However, the study of both properties is the basis for creating a new concept of personalized and sustainable product. In accordance with this approach, a conceptual design procedure of packaging with personalized and adapted geometries based on the digitization of fresh food is proposed in this work. This study is based on the application of advanced technologies for the design and development of food packaging, apples in this work, in order to improve the quality of the packaging. The results obtained show that it is possible to use advanced technologies in the early stages of product design in order to obtain competitive products adapted to new emerging needs.

## 1. Introduction

During the last decade, the demand for healthy and fresh food, especially fruits and vegetables, was gradually growing in a context where eating and consumption habits were constantly changing due to the society lifestyle. For this reason, the European Union raised new objectives for the food packaging industry: sustainability of raw materials, minimization of waste, reduction of energy consumption during the production process, minimization of environmental impact, recyclability of packaging and littering reduction [1,2,3]. 

Besides, consumers demand specific needs, mainly related to the design and adaptability of packaging [4]. Among others, providing more nutritional information on the packaging, greater food security and less risk to health are requested [5,6,7]. Thus, there are studies that place the packaging of fresh product as the second reason for choosing a product where factors such as comfort, appearance, transparency and texture should be considered [5].

Consequently, regarding the selection of material, thermoplastic polymers comply with all of these conditions, which is why they have been chosen by manufacturers of different products as materials for their packaging [8]. Polyethylene terephthalate (PET) is a polymer whose properties include its mechanical resistance to both impact and chemical products, its transparency, its lightness, the reduced demand for energy in its manufacture and transport, its mouldability and its recyclability [8]. In addition, it is the most recycled plastic in the world, and the European Food Safety Authority has corroborated that PET does not contain bisphenol-A (BPA), phthalates or dioxins [2].

However, PET has been identified as one of the main causes of global environmental degradation and this problem is expected to increase due to growing demand from developing or re-industrializing countries around the world [9,10,11]. For example, China, which is one of the largest exporters as well as producers, accounted for 24% of the world’s demand for PET plastic in 2016. For this reason, understanding the consumption needs of this type of market is critical when examining the prospects for global PET volume growth [12].

In fact, the clear trend towards the increase in consumption of food that needs packaging carries with it the constant development of materials and techniques that improve the performance of this service [13]. In short, several aspects can be highlighted [14]: the improvement of the production times, the reduction of the material and the improvement of its aesthetic and functional properties. 

Thus, the fresh food packaging industry is interested in developing efficient and innovative solutions to ensure the quality of the products by taking into account their sustainability, especially at the environmental level [12]. For this reason, the design evaluation in the development of sustainable products should include aspects related to the design, manufacture and use of the packaging [15,16].

In this context, the packaging design process implies the consideration of aspects associated with its cost, appearance, usability, manufacturing, sustainability, standards or competitiveness [15]. Therefore, the selection of the manufacturing process of the food package is extremely important and, in this case, one of most commonly used is the thermoforming process [17]. Previous research on the packaging design process in thermoforming focused on the study of the moulds and the materials used in the process [10,18,19,20]. Also, recent research implements functions for intelligent packaging development through which sensitive labels control the condition of the food [21]. These initiatives are aimed at improving food health and freshness, achieving in this way its better preservation [4].

On the other hand, the packaging sector demands customized solutions in terms of shapes, sizes and colours, so that each packaging solution is unique [22]. In turn, it is intended to pursue product differentiation by means of sustainable products and new designs [23]. In such a way, it is also possible to respond to demographic changes and consumption habits [24,25,26]. Thus, packaging can be personalised in design, brand and/or size, among other characteristics. Also, it can be understood that an adapted packaging is the one that adapts itself to the inner shape and size of the product [27].

However, despite the needs that were determined in previous research and after having analysed the fresh food packaging which is currently commercialised on the market, it is observed that the majority of them are standard and just a few include customization in terms of forms [28]. In addition, there is no adaptation to specific sizes according to calibres in these cases.

For this reason, this work studies the personalization and adaptation in packaging design for the protection of the product in order to provide designers with a tool for the packaging 4.0 generation. This is linked to the sustainability of both the interior of the product and the material expense. A case study with apples is shown in order to validate the use of advanced technologies as part of the unconventional design of packaging, increasing the sustainability and functionality of these products. Thus, a technique that allows the design of personalized packaging to optimize the cost of packaging material, the technological resources and to improve the functionality of packaging is developed. The research also focuses on obtaining packaging that offers brands the possibility of differentiating themselves by means of personalization and in accordance with sales expectations and consumer perception.

## 2. Materials and Methods

### 2.1. Tools and Materials Used to Obtain the Customized Packaging

To carry out the experimental development, apples were used as the target product because their size facilitates the acquisition of measurements, studying the adaptation to packaging depending on two different calibres: category I and category II, according to [29].

**Figure 1:**
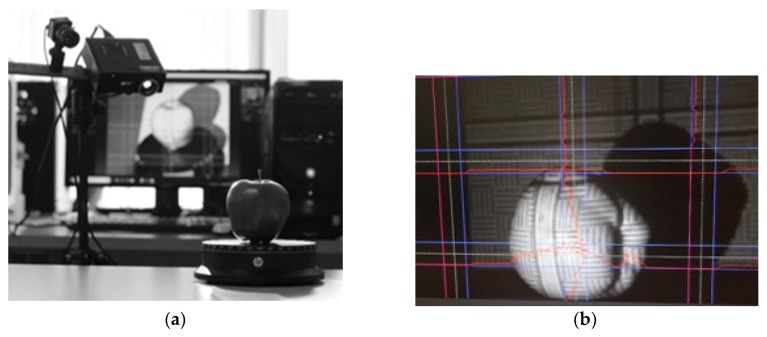
(**a**) Scanning procedure using the SLS-1 David^®^ V5 scanner, (**b**) Visualization of the apple through the software (HP 3D Scan David^®^, version Pro V5, HP inc., Palo Alto, CA, US).

**Figure 2:**
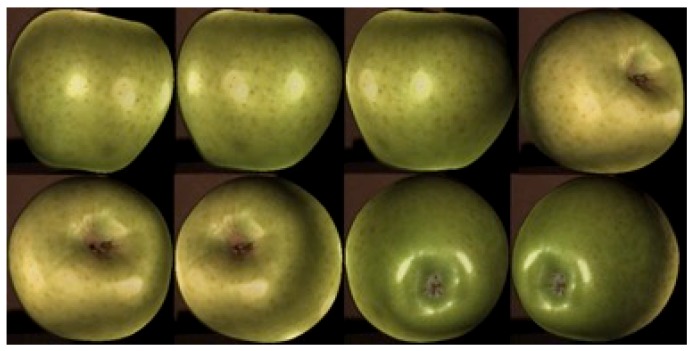
Example of image capture, which the 3D scanner performs, of an apple of calibre 1.

For the development of the experimental procedure, 10 units of each calibre were used. To carry out the design-adapted concept from the computer-aided design (CAD), the apples were digitized by means of 3D techniques [30], Figure 1. To this end, 8 images were captured for each unit to create a three-dimensional model, Figure 2. An SLS-1 David^®^ 3D scanner (David Vision Systems GmbH, Koblenz, Germany) of Hewlett-Packard (HP inc.) was used, according to [31]. These scanned elements have, as their main purpose, the creation of adapted geometries through the generation of curves based on the scanned elements. 

Thus, the concept development was carried out through CAD software, Solidworks^®^ (2016 version, Dassault Systèmes SE, Velizy-Villacoublay, France). As a result, the development of packaging design has been simplified in time and has allowed us to generate more complex and personalized forms for the food.

**Figure 3:**
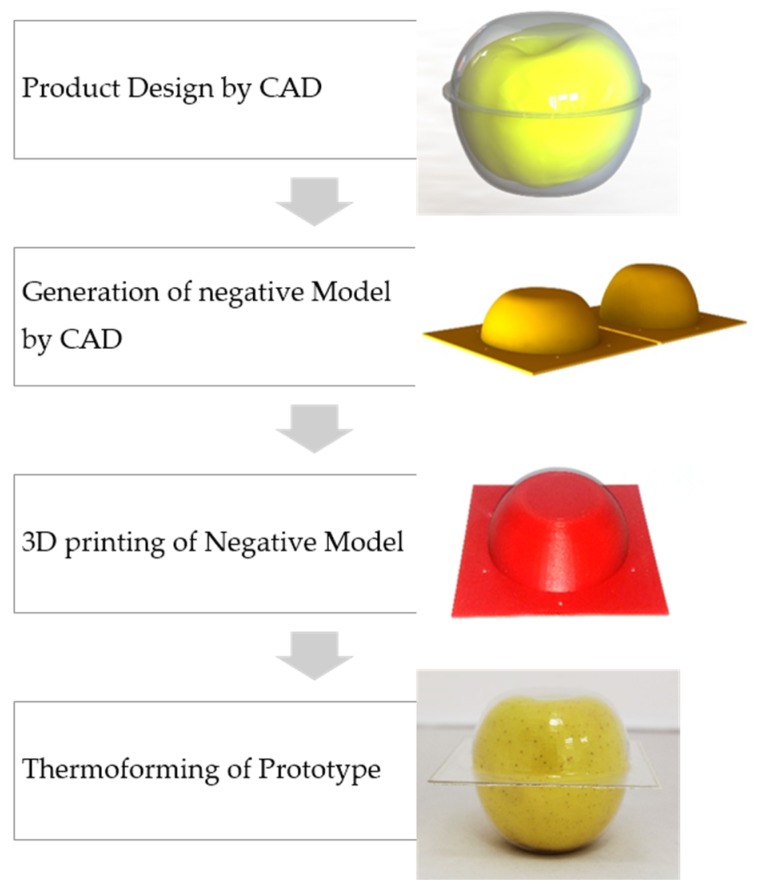
Generation of prototypes in thermoforming with moulds created by FDM.

The evaluation of the design proposals was validated through the generation of reliable prototypes. The mould design and The Standard Triangle Language (STL) file of the mould were generated by Solidworks^®^ and were then parameterized using software for 3D printing, Simplify3D^®^ (Cincinnati, OH, USA). A Gcode file was generated for printing in the FDM machine BQ Witbox (Mundo Reader S.L., Madrid, Spain), that uses a diameter filament of 1.75 mm. A PET sheet of 500 μm thick was thermoformed to generate the prototype with Formech 450DT using the FDM mould, Figure 3.

### 2.2. Parameterization of the Packaging Design

**Figure 4:**
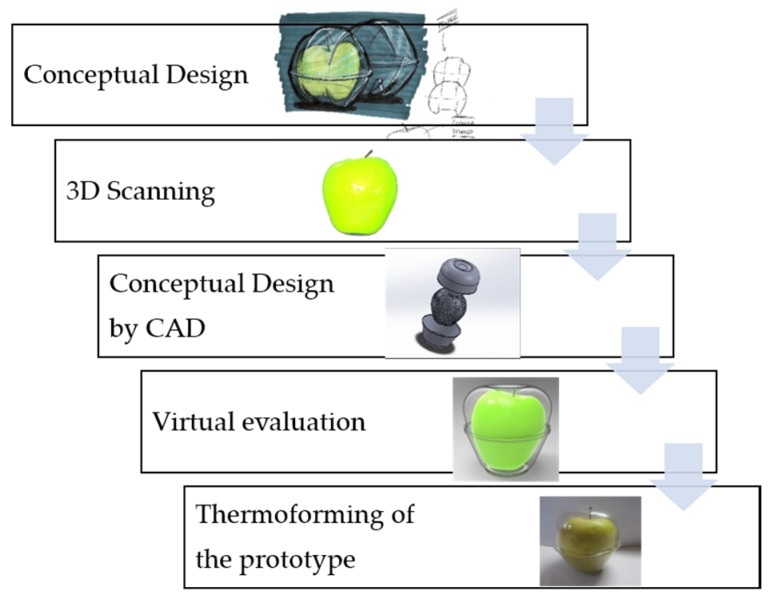
General procedure used to obtain the final package.

Figure 4 details the procedure carried out for the design and development of customised and adapted packaging. Once the conceptual design of the package has been carried out for this case study, the properties of these products are studied through the application to scanned apples, in order to define the main parameters to be taken into account. These data were used for two purposes: packaging design using CAD tools and parameterization of the final geometry. Then, the parameterized package was validated by prototypes made with FDM moulds.

**Figure 5:**
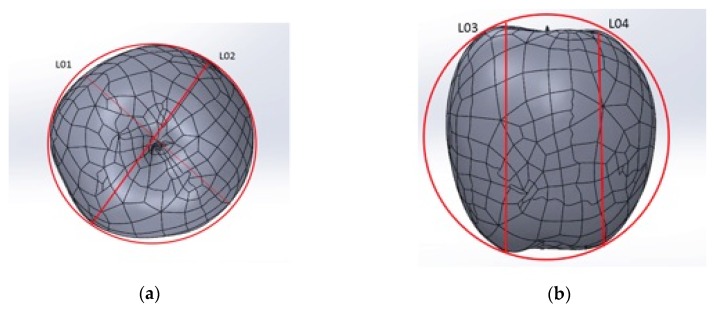
Measurements collected on the digitized products: (**a**) width measurements, (**b**) high measurements.

Also, several functional measures of each natural product were evaluated to analyse the differences between them, also using Solidworks^®^ software. These dimensions were selected according to the parameters established for the calibres: the largest diameter, Dm, and the maximum height, H. According to this, two measurements were collected for each of the dimensions studied: L01 and L02 for Dm and L03 and L04 for H, Figure 5. It should be noted that L01/L02 were made in the two directions of the maximum diameter of the fruit and L03/L04 for the two highest recorded heights. This allows the calculation of the variation of the measurement between pieces of fruit.

One of the objectives of digitizing these elements, because of the normal variations of a natural product, is to define the range of measures that present representative variations that must be taken into account for the design of the packaging. As a result, comparative tables were obtained and the dimensional range was defined, which will serve to obtain the adaptation parameters on the design of the packaging, which is the object of study.

Once the concept was generated, the design was developed in Solidworks^®^ using the 10 scanned elements as a means of generating the construction curves. In accordance with this, the set of lines and tangent arcs, which together form the design of the idea previously conceptualized, were defined. Likewise, the numerical relations between the different container geometries were defined, using as relation parameters the maximum height, H and the maximum diameter, Dm, of the container.

Then, when the final design was developed in Solidworks^®^, the relationship equations, defined above, were introduced in order to evaluate the degree of adaptability through this type of digital tool. Then, the variable measures of the packaging were defined in order to carry out the adaptation to the two categories of size of apple studied according to [31].

**Figure 6:**
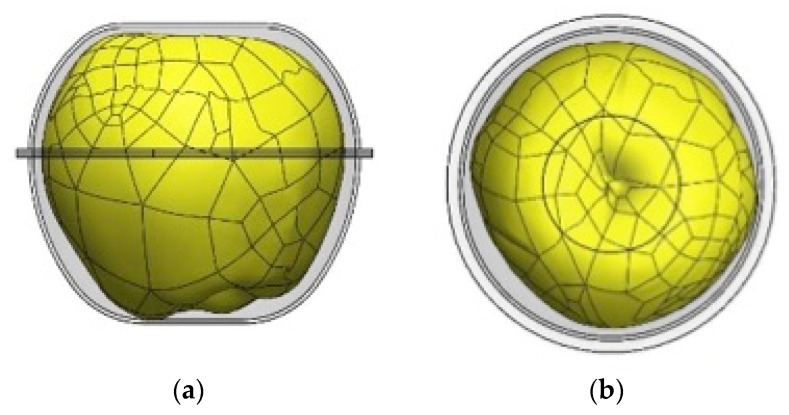
Example of the result of the adaptation parameters with respect to the digitized product: (**a**) evaluation of the high packaging designed, (**b**) evaluation of the width of the packaging designed.

Finally, the adaptability range of the design created for this practical case was established and, in addition, the degree of adaptation of the dimensions obtained with each of the digitalized units for the two calibres studied were evaluated. Figure 6 shows an example of the results obtained.

The two calibres studied were evaluated through the creation of a reliable physical prototype using FDM moulds for thermoforming of the sheet. The developed prototypes were then used to validate the dimensions obtained by introducing real apples. In total, 20 apples were used for each calibre at this stage and the selection of each of them was made taking into account shapes and size variations. Also, different types of varieties were included.

## 3. Results

### 3.1. Virtual Evaluation of Digital Elements

**Figure 7:**
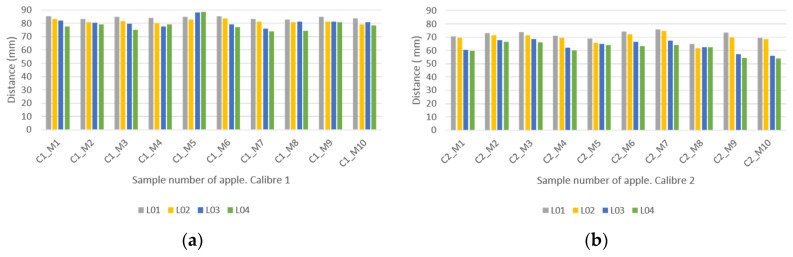
Measurements collected from scanned parametric models of apple samples: (**a**) calibre 1, (**b**) calibre 2.

As described above, the study was conducted for two types of calibres, according to [29]. As seen, the calibre refers to the predominant size within the packaging and is defined according to the maximum equatorial diameter [32]. Thus, the apple samples were scanned to generate a parametric model of each one to make the measurements according to the methodology. The data obtained from the samples studied are detailed in Figure 7.

From the results obtained from the study of the morphology of apples it is determined that the dominant geometry of the fruit is slightly oval. Therefore, a major axis, L01, and a minor axis, L02, can be defined in order to name the maximum dimensions of the digitized samples. It is worth mentioning again that L01 and L02 correspond to the average dimensions of the width of the apple. 

**Figure 8:**
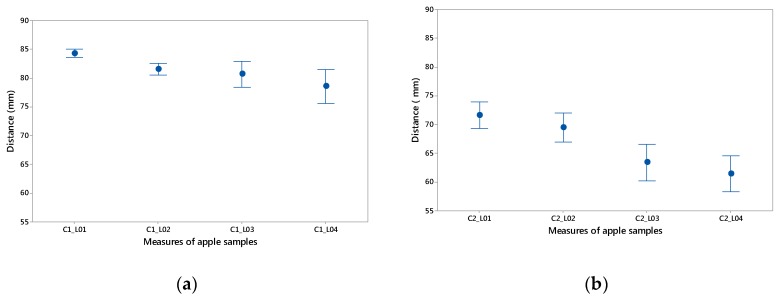
Mean data for parameters L01, L02, L03 and L04 with measurement dispersion: (**a**) calibre 1; (**b**) calibre 2.

On the other hand, L03 and L04 are the height measurements collected on the digitized elements. Figure 7 shows that, generally speaking, calibre 1 (category Extra) is larger than calibre 2 (category I). For calibre 1, parameter L01 lies between 83 mm and 85.5 mm, while L02 varies between 80.2 mm and 83.75 mm, Figure 8a. This shows the disparity of measurement for apples of the same calibre, intrinsically affecting their standardisation for subsequent parameterisation.

Regarding the height of the apple, for parameters L03 and L04, the aim is to find the maximum per calibre of the apples. In this case, L03 is the larger dimension with measures between 77.7 mm and 88.16 mm.

As for calibre 2, in Figure 8b, the measurement intervals tend to increase according to standard (CE) N° 85/2004 [29]: 5% for calibre 1 and 10% for calibre 2. As for the measurement results and because parameter L01 is predominant from the point of view of packaging design, a measurement range between 69.4 mm and 75.9 mm is observed. Likewise, as far as the maximum height is concerned, parameter L03 also plays a part in the design and varies between 56 mm and 68.5 mm.

On the other hand, Figure 8 shows the mean data for the four parameters with their standard dispersion. In both cases, the measurements show a greater dispersion for the parameters L03 and L04, being slightly higher for the apples of calibre 2. L01, which has a greater influence in the design of the package, exhibit more homogeneity than the other dimensions.

Analysing the data presented so far, it can be deduced that the sizes of the packages of a certain calibre can be grouped in a dimension that encompasses all sizes of apple inside a category, including the difference between all the fruits of the same type and calibre. Therefore, the variations in the samples that affect the design of the packaging are the maximum width and height obtained from grouping the digital models.

**Figure 9:**
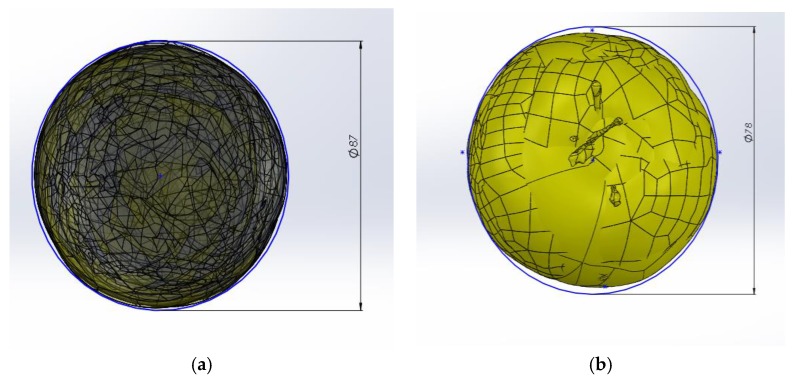
Overlap of the 10 apple units to establish a common diameter based on the sample: (**a**) larger size 1: (**b**) size 2 with dimensions between 63 and 73 mm.

For calibre 1, the maximum measurement within the calibre established according to the standard and with a tolerance of 5% [29] was 85 mm. For virtual measurements, a maximum diameter of 86.7 mm was obtained, Figure 9a. For calibre 2, with a maximum nominal size of 73 mm and a permitted tolerance of 10% according to [29], 78 mm was obtained as maximum diameter for the virtual measurements, Figure 9b. These dimensions served as starting points for the dimensional study of the container, although these dimensions could be reduced due to the irregular geometries.

Although all apples are included in the same diameter, the plant shape is slightly oval, as discussed previously. Thus, the linear dimension on the plant in one of the sides is lower with respect to its perpendicular components, L02 and L01 respectively. According to this, a dimensional relationship, Dr, was established for the diameter, according to Equation (1):

(1) $$\mathrm{Dr}=\frac{L01}{L02},$$

**Figure 10:**
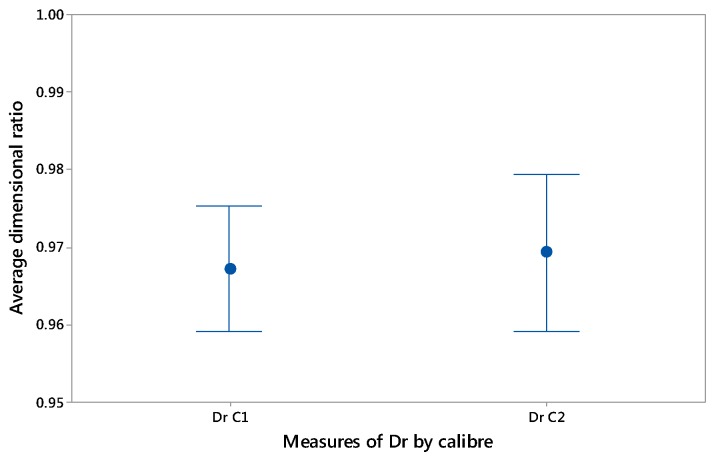
Average dimensional ratio between dimensions L01 and L02 for calibre 1 and 2.

Thus, from the data obtained in the 20 case studies corresponding to calibres 1 and 2, a dimensional relationship was established between both distances of 0.97 and 0.98, respectively, Figure 10. This relationship was used for the design and parameterization of the packaging by CAD, giving rise to the final geometry of a non-cylindrical container.

It is important to bear in mind that these dimensions were studied at an experimental level. The aim is to propose a method in which, using scanned elements, designs can be generated. To this end, a clearance coefficient, that can be applied to the dimension of a given calibre, Cd, was proposed. 

Analysing the dimensional results of the three-dimensional models of the scanned apples and the measurements according to the norm, a particular Cd can be defined for the design of a packaging. This coefficient was defined as the ratio between the observed maximum dimension per calibre according to the norm, and the virtual dimensions studied. Consequently, this definition makes it possible to ensure that the size of the containers is adapted to all geometries included within a calibre.

As an example, if, for calibre 1, the maximum diameter allowed is 89.25 mm including tolerance, and the maximum diameter that appears as a measure of nominal calibre is 85 mm, then Cd could lie between 1.05 and 1.06. However, because of the differences that naturally arise in fresh foods, 1.06 was considered for use to ensure proper functioning. Thus, the validation of this coefficient was carried out by implementing this coefficient in the development of the adaptation of calibre 2.

Therefore, the calculation of packaging dimensions can be done according to the following equation:

Dm = Cd × Cm,(2)

where Cm is the maximum nominal size of the calibre, Dm is the width of the packaging and Cd is the clearance coefficient.

Then, for designing the packaging for calibre 1, Dm is 90 mm; this size is obtained by multiplying 1.06 and 85 mm. In the case of calibre 2, Dm is 77.4 mm, obtained by the multiplication 1.06 and 73 mm. These data were validated during the parametric design, which is explained in the following section.

The norm does not specify apple heights by calibre. Therefore, from the measurements made on the scanned samples, a ratio, Rd, was established between the average dimensions of width (L02) and height (L03) of the apples, Equation (3).

Rd = L02/L03,(3)

Therefore, the Rd for calibre 1 corresponds to 1.05 and 1.07 for calibre 2. This means that, compared to calibre 1, calibres 2 have lower height in relation to their diameter. Then, the total height H of the package in this case study is given by Equation (4).

H = Dm/Rd,(4)

Thus, in the parametric design of the package, Cd is implicitly included in all the dimensions of the package from the Dm obtained in Equation (4). It should be stressed that this ratio, Rd, can be modified to obtain containers with different height in relation to the width.

### 3.2. Parameterization of the Conceptual Proposal

**Figure 11:**
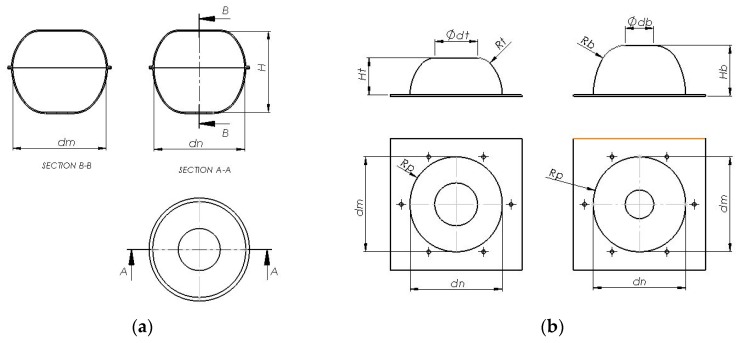
Dimensions of the container and the mould: (**a**) Plan of the variables that affect the container and therefore the upper and lower mould; (**b**) Dimensions of the moulds, on the left upper half and on the right lower half.

Once the design of the packaging was carried out, two variables, the maximum height of the container, H, and the maximum width, Dm, were used to define a series of parameters that characterise the package. Figure 11a shows a diagram of the variables that affect the sizing of the container.

From the initial geometry extracted from the concept design of the apple, the packaging was parameterized according to a series of equations described below. Figure 11b shows a diagram with the dimensions that affect the mould when thermoforming the designed package. Thus, the equations that affect the overall dimension of the packaging correspond to:

Hb = H × 0.57,(5)

Ht = H × 0.43,(6)

Dn = Dm × 0.97,(7)

where Hb is the height of the bottom half of the designed packaging and Ht is the height of the top half. In the same way, the projected dimensions of the container are given by the greater width, Dm, and the smaller width, Dn. In this case study, an oval geometry has been created.

As mentioned above, the design created in this study consists of a series of arches that are tangentially joined, in plan and profile, and a flat surface at the ends with a circular shape. The curves that define these geometries, Figure 11, could be related by means of equations from the initially created CAD design. Thus, the relationship between H and the slightly oval curved geometry that makes up the package design in the profile view is given by the equations:

Rb = H × 0.27,(8)

Rt = H × 0.18,(9)

db = Dm × 0.3,(10)

dt = Dm × 0.45,(11)

Rp = (Dm + 10)/2,(12)

These equations define the radius of curvature of the upper part, Rt, and lower, Rp. In addition, the adaptation of the dimension of the flat part, so that the packaging can be easily supported, is given by the diameter in both halves of the container, db and dt, and was related to the parameter dm. On the other hand, the radius Rp is the parameter that encompasses the overall geometry of the container in plant, and the shape was constructed by sweeping through the vertical curves given by Rb and Rt.

The rest of the tangent arcs that make up the packaging design are automatically adapted from the curves generated with the above equations.

Then, the generated equations were introduced in the parametric design software, Solidworks^®^. The benefit of the parametric design of a packaging is the customization of the geometry according to the need of adaptation to the product to be contained. In addition, the design can be evaluated in real time by means of the digitized fruit samples. As a result, all construction operations such as roundings, tangent arcs, etc. were related. The number of operations obtained was a total of 12 for each part of the container. These equations and variables serve to quickly modify the geometry of an object.

For the lower part, according to the equations defined in the previous section, a relationship was defined between the curves that make up the container and, therefore, the mould. These equations were related to the sketches made in the parametric design program. In the same way, the equations of the upper part were parameterized.

In short, by modifying one of the measures, the packaging is automatically adapted. This is one of the first steps for the generation adapted to a specific packaging design in the context of industry 4.0.

**Figure 12:**
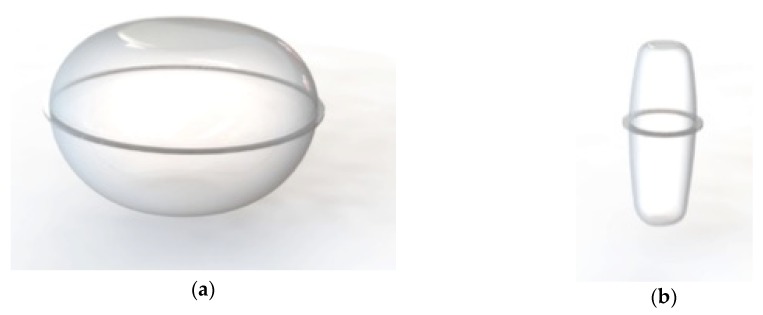
Example of maximum and minimum ratio between Dm and H for H equal to 85 mm: (**a**) Maximum ratio for Dm = 137 mm; (**b**) Minimum ratio for Dm = 20 mm.

Another result obtained in this case study is the maximum and minimum ratio (Rd) of measures between H and Dm, obtaining a range of adaptation measures. The maximum Rd is 1.32 and the minimum is 0.24. This range of measures means that the packaging created can also be used for other types of fresh food or measurements. Figure 12 shows an example of the variations in function of the maximum and minimum ratio.

Also, if Dm is kept constant in the two parts, top and bottom, of the parametric packaging, Ht and Hb can be adapted independently, in the CAD file, to obtain intermediate H measurements by modifying the H value only in one of the parts. Also, with this modification, Rt and Rb also adapt automatically according to Ht and Hb, respectively. This parameter offers greater versatility if possible combinations for containers using fewer moulds are taken into account.

Once the packaging was parameterized, a series of configurations were established in Solidworks^®^ software to evaluate the appropriate sizes for each of the samples studied and with which the theoretically established Cd was validated. The configurations allow the packaging to adapt automatically to the measures established, making it easier to adapt to calibres and measures according to specific needs.

### 3.3. Evaluation of Results

In order to analyse the viability of the coefficient obtained, Cd, using the 3D scanned fruit models, the correct arrangement of the apple was evaluated with respect to the dimensions of the packaging. Then, after analysing the dimensions with the configurations of the packaging, it was observed that the Cd corresponds adequately, although the optimum height for each sample varies, Appendix A.

Bearing this in mind, it can be concluded that, depending on the design needs for the same calibre, several containers with different H could be constructed. This may be possible thanks to new technologies such as additive manufacturing, which allows low-cost moulds to be made in order to optimise the maximum performance of the container. However, if the design requirements allow it, with this methodology it is possible to establish an optimal design that encompasses all food units of the same topology.

On the other hand, generating a custom packaging for a unit is also possible. This example could have multiple applications that could be extended to other types of products.

Thus, the final dimensions, which were adapted to the size obtained according to the Cd calculated in the measurement part, correspond to calibre 1 with the ratio Rd of 1.05, with Dm of 89.3 mm and H of 85 mm. Although, as it was mentioned, some of the samples studied could modify the H for the use of the dimensions having a range between 80 mm and 85 mm. Similarly, for calibre 2, the dimensions correspond to the ratio Rd of 1.07 with Dm and H being 75 mm and 70 mm, respectively, and, according to the samples studied, with a range of H between 67 mm and 70 mm. For more information see Appendix A.

**Figure 13:**
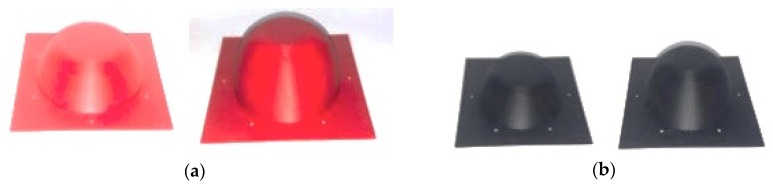
Parts, inferior and superior, of the mould to generate the physical prototypes: (**a**) Moulds for calibre 1; (**b**) moulds for calibre 2.

Finally, the results obtained for the two calibres studied were validated by means of a physical prototype. The FDM moulds created for the two calibres are shown in Figure 13.

**Figure 14:**
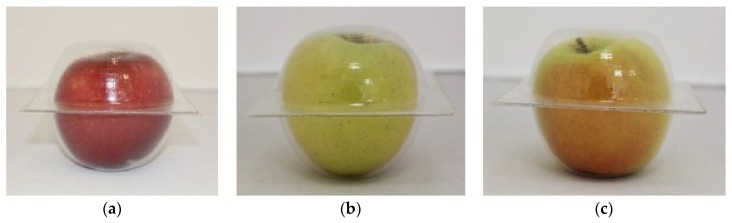
Images of the evaluation carried out with the prototype and commercial fruits: (**a**) Pacific rose apple, (**b**) Golden apple, (**c**) Jonagold apple.

The design was validated using the physical prototypes and 20 apples for each calibre studied, Figure 14. These apples were selected taking into account shapes and size variations, and different varieties were included in the units studied. For more information see Appendix B.

Once the results were analysed, it can be said that the final solution obtained is positive as all the units of the same calibre correspond to the dimensions of the packaging. However, according to the studies in the parametric software, it can be seen that, in several of the cases studied, the dimensions are not adjusted in their entirety, causing parts of the packaging to be empty. This is not a functional problem for the design because these variations are given by the wide range of measurements that are included within a calibre. Specifically, these dimensional deviations are accentuated in calibre 2, which is a smaller calibre and comprises a larger range of measurements. In the case of calibre 1, which belongs to category I according to [28], lower dimensional deviations are observed. However, the morphological inequalities between the different apple varieties are larger.

In this situation, personalisation and adaptation could be increased by reducing the spectrum of fruit types that can be introduced in the same type of packaging. Therefore, depending on the type of product to be packed, adaptation and personalization can be considered with a greater degree of accuracy.

## 4. Discussion

The main idea of reverse engineering is to synthesize a fruit model so that it can be used and measured as part of the design process. The studies carried out show that it is possible to generate personalized designs and that they can also be adapted according to the specifications required by a specific product. Digitization and flexible designs make it easier to customize and test concepts in real time to help design teams make faster decisions and with greater reliability [33].

Then, the introduction of scanned elements in the design process facilitates the adaptation of the size and shape of the packaging to the type of fruit contained, allowing only the quantity of plastic material necessary for its sale and transport to be used. In this way, the product is optimised, which means a reduction in environmental and economic impact. This fact is relevant because, currently, thousands of tons of plastic and food are discarded daily [12], and great efforts are made to mitigate this impact, generating stricter and stricter regulations in the withdrawal and recycling of food packaging [34]. The huge quantity of packaging manufactured means that a small reduction in each container has a great influence on the environment. This working methodology promotes the reduction of material that is so necessary in a strategic sector such as the food industry. Thus, by adapting the packaging to the size and shape of the food, less raw material is wasted. In addition, the technique presented in this paper for designing efficient packaging also offers the possibility of creating packaging with less food, adapted to emerging consumption needs, which translates into less food waste and, therefore, in the protection of the environment. In addition, this tool can be used with any type of sustainable raw material, i.e., PLA (Polylactic Acid) sheet, although the material currently on the market has been used in this work.

As noted above, much of the food waste is produced by using containers with large amounts of food that end up deteriorating in homes [35]. Thus, the possibility offered by this methodology for the realization of custom packaging by type of fruit and category can lead to the reduction of food waste due to the possibility of reducing the amount of product inside and by improving the preservation of specific properties of each food [36].

The application of digital elements in the design process was validated as part of the creation of a method that includes advanced technologies in its procedure, as was researched in other fields of engineering [37,38]. In the study, two main parameters related to the functional measures for the containers were selected, the maximum height and width, to adapt a packaging to a given calibre. However, the geometry of a fresh food is irregular so four measurements at the top and bottom were taken to calculate the height and width. It should be added that these measurements had been carried out to analyse the dimensions of the product studied at laboratory level. The main objective of this work was to evaluate the direct application of the scanned models for the generation of personalized packages for a range of measures established within a calibre.

This study proposed a 3D scanning scheme for fresh food to support custom packaging design [31]. Then, the three-dimensional model of the fruits approximates the real geometry. Thus, the application of 3D fruits facilitates the realization of personalized and adapted designs, as well as the evaluation in real time of design proposals. Moreover, the parametric design of the package according to the two parameters studied, gives rise to the possibility of generating packages adapted to the dimensions and needs of the food according to its established range of measures. In short, the result was the creation of a packaging, fully defined by equations, which is capable of adapting to the measurements in a given range.

Based on these results, computational design is aimed at the parameterization to favour customization by means of optimal configurations of the variables, favouring the adaptation of the design to specific needs [39,40]. Computer programs facilitate the realization of personalized and flexible designs to adapt to specific needs by means of the parameterization and digitalization of elements [41,42,43]. Product customization also serves as an engine to improve sustainability throughout the product life cycle [44]. Custom design enhances product design by meeting the specific needs of users [45].

Furthermore, according to new trends and competitiveness, the design and development of packaging need solutions that streamline the working procedure. In this context, based on the approaches made on the customization of packaging, there is also a latent need for quick and flexible solutions. Specifically, the process of manufacturing moulds by conventional methods usually delays the validation time of the final prototype of the product, so designers cannot make changes or explore quick and reliable options [46]. Facing this situation, the application of additive manufacturing techniques can provide design teams with a fast and economical tool that can thus be used from the early stages of product design.

Summarizing, it is possible to build reliable prototypes by additive manufacturing. The similarity of the prototypes generated thanks to 3D printing and thermoforming technologies shows the possibility of creating prototypes that provide greater reliability in a simple way. Then, the evaluation by means of these prototypes provides a trustful tool for the validation of the mentioned designs. In short, it was proven that it is possible to thermoform geometries with different shapes. Therefore, it is possible to obtain products with better performance and, consequently, competition. This can also affect the life cycle of the product as there is a significant improvement from the point of view of the social, economic and environmental impact.

Finally, this work presents several future lines of action, the most important of which is the advanced study of new packaging designs by means of topological optimisation and the extension of the study of virtual environments for early evaluation.

## 5. Conclusions

The following conclusions can be drawn from the research work carried out in this study:

It was possible to digitize fresh food by means of 3D scanning techniques, obtaining reliable digital elements that could be used in advanced technologies. In this sense, it was possible to define a procedure for the reverse engineering of different types of food, detailing the specific parameters according to size and finish.

It was possible to use the digitized fruits during the conceptual phase in the packaging design process. Thus, custom designs were developed using these elements as a reference during computer-aided design, thus validating the proposed methodology. In addition, the evaluation of the ideas generated was also favoured by the possibility of checking dimensions in real time using these digital products. On the other hand, the adaptation according to the specifications required by a specific product was also improved. Therefore, the application of 3D fruits facilitated the development of customized and adapted designs.

It was possible to completely parameterize the geometry of the package to create custom and, in turn, automatically adaptable designs. In short, the creation of a packaging, fully defined by equations, was able to adapt to measurements in a given range. The parameterized design was made possible by means of the virtual evaluation of digitized fruits.

Finally, by adapting the containers to the size and shape of the food content, less raw material used for the container was wasted. Likewise, the technique presented in this work to design efficient containers also offered the possibility of creating containers with less food, adapted to emerging consumption needs, which lead to less food waste and therefore a better protection of the environment. In addition, this tool could be used with any type of sustainable raw material, although in this study the material currently on the market was used.

## Appendix A

**Table A1:** Images of virtual evaluation for both calibres.

Sample	Calibre 1		Calibre 2	
	Front View	Top View	Front View	Top View
M1				
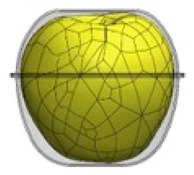
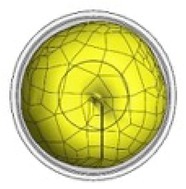
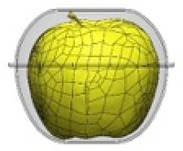
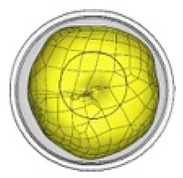
M2				
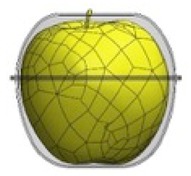
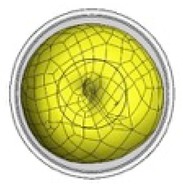
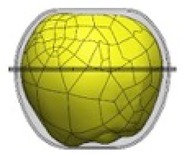
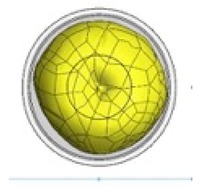
M3				
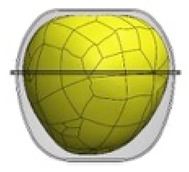
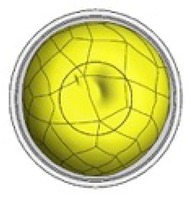
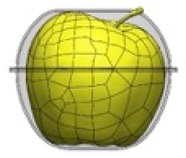
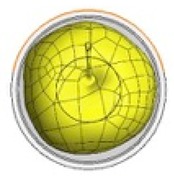
M4				
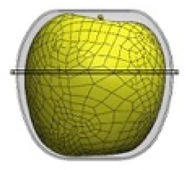
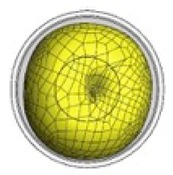
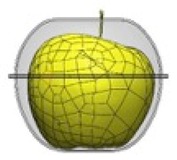
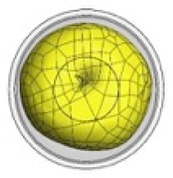
M5				
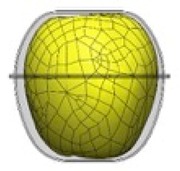
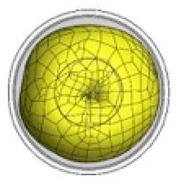
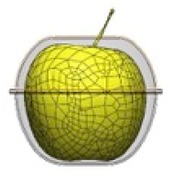
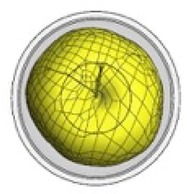
M6				
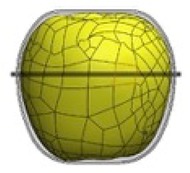
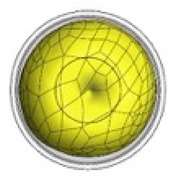
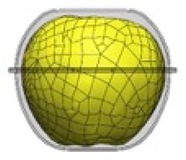
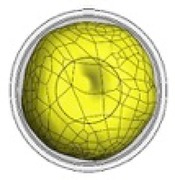
M7				
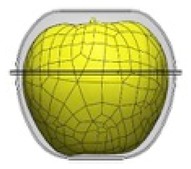
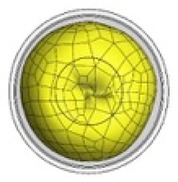
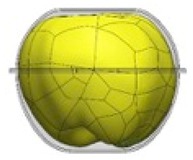
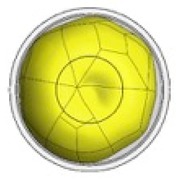
M8				
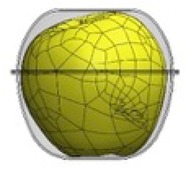
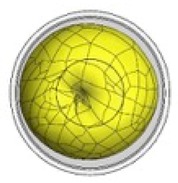
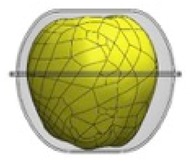
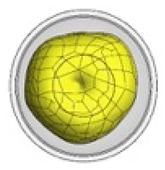
M9				
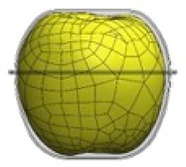
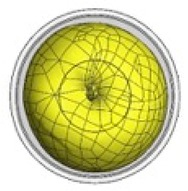
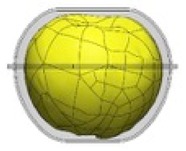
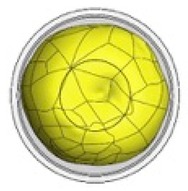
M10				
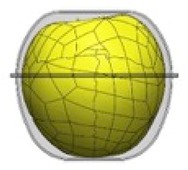
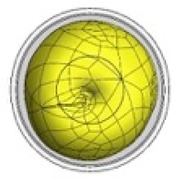
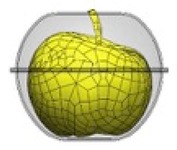
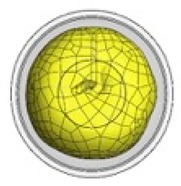

## Appendix B

**Table A2:** Images from the validation study with prototype of the custom packaging for Calibre 1.

Diferent apples inside of designed packaging for Calibre 1			

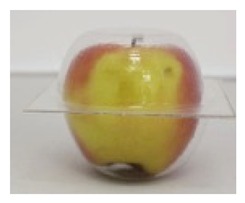
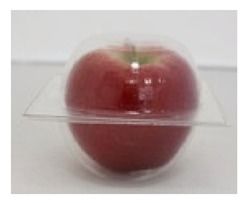
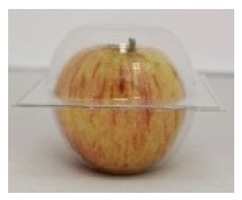
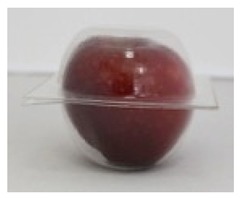

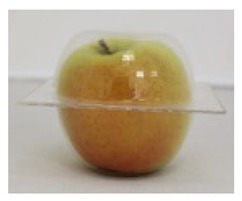
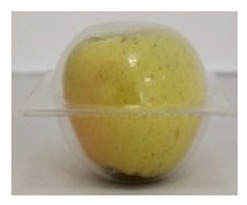
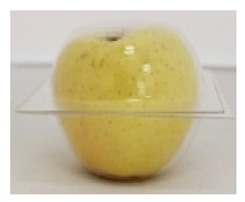
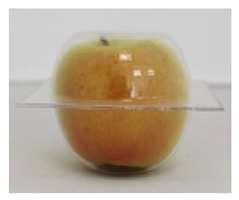

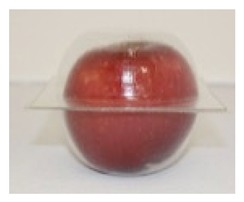
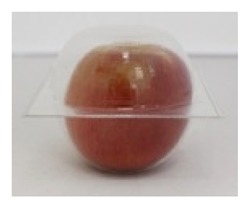
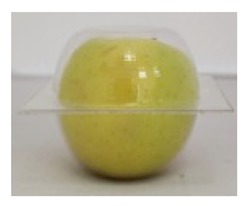
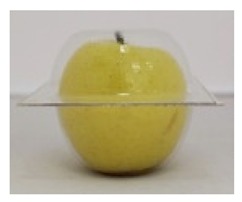

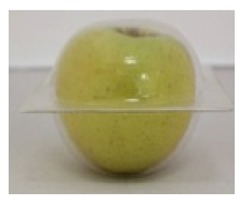
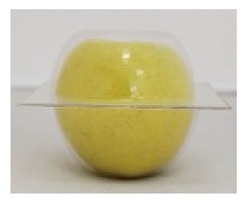
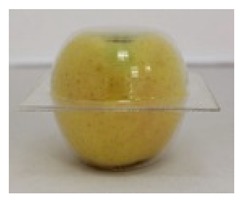
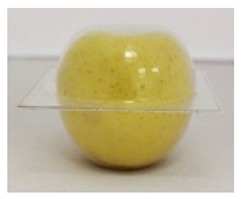

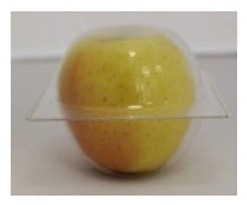
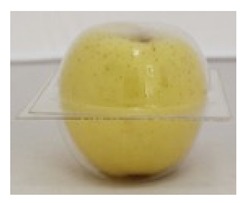
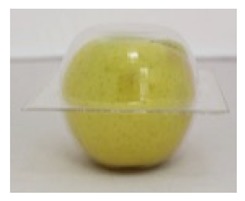
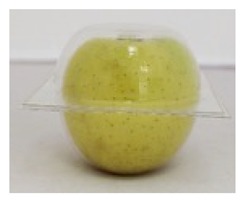
			
			
			
			
			

**Table A3:** Images from the validation study with prototype of the custom packaging for Calibre 2.

Diferent apples inside of designed packaging for Calibre 2			
			
			
			
			
			
